# Music and physical activity in early childhood: the ambiguous role of the at-home context and extracurricular activities

**DOI:** 10.3389/fpsyg.2026.1729705

**Published:** 2026-02-12

**Authors:** Alicia Lucendo-Noriega, Arja Sääkslahti, Alessandro Ansani, Katariina Henttonen, Emily Carlson, Suvi Helinä Saarikallio, Petri Toiviainen, Tanja Linnavalli

**Affiliations:** 1 Department of Music, Art and Culture Studies, Faculty of Humanities and Social Sciences, University of Jyväskylä, Jyväskylä, Finland; 2 Centre of Excellence in Music, Mind, Body and Brain, University of Jyväskylä, Jyväskylä, Finland; 3 Faculty of Sport and Health Sciences, University of Jyväskylä, Jyväskylä, Finland; 4 Department of Education, Faculty of Educational Sciences / Cognitive Brain Research Unit, University of Helsinki, Helsinki, Finland

**Keywords:** at-home context, early childhood, language skills, music education, physical activity

## Abstract

**Introduction:**

This exploratory, cross-sectional study investigates how music and physical activity (PA) engagement at home, and attendance in music and PA based extracurricular activities are associated with children’s verbal fluency (word generation and sentence repetition), inhibitory control, emotion recognition, music and motor skills.

**Methods:**

Participants (N = 103) were from 10 early childhood centers in a Finnish mid-size city and completed the related measurements in the school settings at a mean age of 49 months (SD = 3.5).

**Results:**

A positive association between attendance in music as extracurricular activity and the word generation test was observed. Interestingly, an opposite trend between the reported use of physical activity at home and the sentence repetition score was noted. Lastly, a surprising association was observed as the children with the highest amount of music and PA at home scored the lowest in the emotion recognition measure.

**Discussion:**

Further longitudinal data is needed to explore the preliminary trends observed in this data. Future research should also consider other types of direct-measures and contextual factors. Nonetheless, the results emphasize the need to explore how the early context and experiences might impact children’s development, and how to better support them through diverse activities, such as physical activity and music.

## 1 Introduction

The Finnish National core curriculum for early childhood education and care (ECEC) defines the early childhood period (0–6 years) as a key phase for learning and development (National Core Curriculum for Early Childhood Education and Care, 2022). Thus, understanding the potential role of several factors and activities in children’s development during this stage is an essential question for the research field. In particular, the roles of music and physical activity (“PA”) have received increasing attention in recent years. As previous results are still not fully congruent, this paper aims at investigating the association of music and physical activity engagement in extracurricular and at-home activities with children’s verbal fluency, inhibitory control, emotion recognition, music and motor skills.

The potential of musical or physical activity to support language learning has been a recurring question in both fields (see for example: Alesi et al., 2021; Pino et al., 2023). While covering several language-related skills are out of scope for this article, both structured music sessions (Linnavalli et al., 2018), and the reported use of music at home (Williams et al., 2015) have been shown to influence 4–6-year-olds’ vocabulary skills. The impact of music at home has been documented even in younger children (Papadimitriou et al., 2021), and in children born prematurely (Kostilainen et al., 2024). More specifically, 3-4-year-olds’ rhythmic and synchronization skills were found to be predictors of phonological awareness, and melody perception of grammar skills, with both effects being supported by the reported use of music at home (Politimou et al., 2019). The links between language and music are thought to stem from shared acoustic features processed in the same areas of the brain (Peretz et al., 2015). Regarding physical activity and language skills, the association might be explained by the favorable social and communicative context created by physical activity games (Kamelia et al., 2025) and the embodied approach of enacting words meanings with body movements (Mavilidi et al., 2015). Still, the results on specific vocabulary and verbal fluency skills, which are closely interrelated (Escobar et al., 2018) and the focus of this study, are more ambiguous. Although acute vigorous activity seems to enhance expressive vocabulary skills in healthy adults (Khanna et al., 2025), the evidence from preschoolers is more contradictory. According to one study, the benefits from a physical intervention on expressive vocabulary seemed to be more connected with the social and cognitive components than the physical activity in itself (Olive et al., 2024). In a review of PA-based randomized control trials, no evidence was found for the impact of PA on verbal fluency (Martin-Martinez et al., 2023).

Inhibition in early childhood is defined as a core foundation for later cognitive processes, and refers to the ability to voluntarily control our dominant behaviors (Li et al., 2022; Li, Zhou et al., 2022). Previous evidence on longitudinal music studies and PA-based RCTs suggests positive effects of music and physical activity on inhibition (Degé and Frischen, 2022; Jamey et al., 2024; Morales et al., 2024; Wang et al., 2023). Interestingly, after 14 weeks, Degé et al. (2022) found that the preschoolers in the music group – but not in the sports group—improved their motoric inhibition. This effect might be explained by the need to integrate multiple stimuli when playing an instrument or a rhythm (Rodriguez-Gomez and Talero-Gutierrez, 2022). Regarding PA, recent cross-sectional evidence from examining different types of PA in school children showed that activity in the school breaktimes had the strongest association with better inhibitory control (Watson et al., 2024). In fact, higher levels of cardiorespiratory fitness and time in moderate-vigorous physical activity were associated with improved preschoolers’ inhibitory control (Luo et al., 2023). Diamond (2000) argues how motor and cognitive development are interrelated, with evidence supporting that exercise in childhood can predict better inhibition response later in adulthood, and neural changes can moderate this positive association (Ishihara et al., 2021). However, inconclusive evidence has also appeared from studies comparing music with active control groups (Linnavalli et al., 2018) and in a review of PA interventions (Wassenaar et al., 2020). Further research is indeed needed to clarify the heterogeneity of results and methodological designs (Rodriguez-Gomez and Talero-Gutierrez, 2022).

Emotion recognition (ER) skills englobe inferring the emotional state of others based on a variety of signals from the body, voice and face (Cuciniello et al., 2025; Martins et al., 2021). In children, challenges in ER have been linked to more reactive and proactive aggression attitudes (Acland et al., 2024), showing the crucial value of ER skills for social–emotional development. Understanding what kind of structured activity supports these skills has thus sparked attention. In terms of structured music training, both cross-sectional and longitudinal studies in children fail to reach conclusive evidence (Martins et al., 2021; Neves et al., 2025). In fact, studies from adults suggest that music aptitude is a stronger predictor of vocal emotion recognition than music training (Correia et al., 2022; Vigl et al., 2024). Regarding the relationship between PA and broader social-emotional development, peer attachment and executive functions seem to partially mediate this association (Li, Wang et al., 2022; Wang et al., 2025). The characteristics of the PA pedagogical approach also seem to model this association, as dynamic psychomotor teaching and specific intervention programs have showed positive outcomes (Gil-Moreno and Rico-Gonzalez, 2023). More specifically, a cross-sectional study on rural children found that their motor development, mediated by working memory, were linked to their ER skills (Ge et al., 2024). However, limited evidence from both fields, taken together with the unclear developmental trajectory of ER skills (Cuciniello et al., 2025; Riddell et al., 2024), calls for further research.

The development and definition of musical skills have been theorized by several authors (see for example: Paananen, 2022; Swanwick and Tillman, 1986), and diverse sociocultural factors have been suggested to play a key role from an early age (Lamont, 2016). For example, for melody discrimination skills, after 6 months of active and interactive music class, infants showed preference for tonal music over atonal (Gerry et al., 2012). Furthermore, studying musical exposure at home suggested that infants’ sensitivity regarding musical rhythms seemed to narrow to culture-specific patterns at 12-months of age (Hannon and Trehub, 2005). These examples show the complexity of studying musical development due to its intertwined skills and cultural aspects.

Motor skills are defined as building blocks of children’s motor development and physical activity (Hulteen et al., 2018). Children need fundamental motor skills (FMS) to manage independently in their everyday motor tasks and physical activities (Goodway et al., 2019). FMS develop during early childhood through maturation, practice and physically active playing (Goodway et al., 2019). FMS are typically divided in three categories: balance skills (such as balancing on one leg and jumping sideways), locomotor skills (like standing broad jump and skipping) and object control skills (for example, throwing and catching a ball) (Hulteen et al., 2018). These skills are recognized as a basis for health enhancing physical activity leading to holistic wellbeing in later life (Stodden et al., 2023). Earlier studies have shown that cognitive functions, social–emotional skills and academic skills are supported by physical activity and motor skill training (Martins et al., 2024).

## 2 Materials and methods

The present study follows a cross-sectional exploratory design, investigating if earlier music and/or sports related activities are associated with 4-year-old children’s skills. This association is examined for both extracurricular activities (sports referred from now on as “physical activity-based”) and, the reported use of music and physical activity related activities in the home environment. While the study is positioned within a post-positivist paradigm involving quantitative and predictive methods, it acknowledges the complexity of discussing children’s development and that any possible observations are also context dependent. The research questions (RQ) set are:

1. RQ1: How does previous attendance in music and physical activity-based extracurricular activities associate with children’s verbal fluency, inhibitory control, emotion recognition, and music and motor skills?
2. RQ2: How does the reported use of music and physical activity at home associate with children’s verbal fluency, inhibitory control, emotion recognition, and music and motor skills?

The data presented in this publication constitutes the first data collection point of a larger project called MUSPRO. For a more detailed information on the study design, refer to the study’s preregistration: https://doi.org/10.17605/OSF.IO/7CK49.

### 2.1 Sample

All caregivers of children born in 2019, and from the recruited municipal early childhood and care (ECEC) centers, received the study’s information flyers, privacy notice and informed consent. The caregivers were not informed beforehand whether the daycare center was receiving music, physical education, or continuing with their business as usual to avoid interfering with participation. Caregivers who were interested in participating, signed the informed consent. The present study was approved by the University of Jyväskylä Humans Science Ethics Committee, complying with the committee’s guidelines and the Helsinki Declaration.

Participating children (*N* = 116) were then students of the ECEC centers, born in 2019 with no other inclusion criteria set. The recruited public ECEC centers were all in a Finnish mid-size city. In Finland, children are usually given a place to one of the closest educational ECEC/schools to their home. Thus, before contacting the potential ECEC centers to participate in the study, the neighborhoods as well as their physical premises were taken into consideration so that there would be similar conditions across all centers and the sample of participants would be representative of the city’s population.

At the start of the data collection, the children’s mean age was 49 months (SD = 3.5). The education level averaged over both caregivers (when applicable) was M = 2, SD = 0.75, min = 1 max = 3, the mean reflecting bachelor’s degree or equivalent. It is important to note that in the present analysis, the participants (*N* = 13) with a different mother tongue than Finnish were excluded. This was not done based on any socioeconomic reasons, since the level of education between caregivers was not significantly different between Finnish and non-Finnish speaking families, but because most outcome measurements are heavily language dependent and some comprehension issues were detected during data collection. As the language of all ECEC centers was Finnish, no exclusion criteria for the further project and analysis were implemented as the longitudinal aspect of the project allowed for language improvement across time.

### 2.2 Procedure

All measurements were conducted on ECEC premises, during the everyday schedule, predominantly in the morning. Research assistants were trained, and all measurements were piloted before data collection. Before tests, children were asked for their verbal assent.

### 2.3 Materials

#### 2.3.1 Background information

The caregivers of the participating children received a background questionnaire asking for aspects such as the age of starting to speak, languages spoken at home, caregivers’ education, as well as their use of music and physical activities at home. The latter questions were: (1) *how often do you sing together with your child,* (2) *how often do you play an instrument together with your child,* (3) *how often do you exercise (low intensity) with your child,* (4) *how often do you exercise (high intensity) with your child.* The questions were answered on a scale from 1 to 5 (1 = never/hardly ever, 2 = monthly, 3 = weekly, 4 = several days a week, 5 = daily). The low and high intensity physical activity were explained in the questionnaire by using examples. The caregivers were also asked to report all the child’s extracurricular activities, detailing the month and year of starting and (if applicable) stopping the activity, as well as the weekly duration of these activities.

#### 2.3.2 Measurements

Table 1 describes the measurements that were used in relation to each of the skills included in this study. These measurements were chosen for the whole duration of the project, and thus, needed to be sensitive to developmental trends during the upcoming 2 years.

**Table 1:** Description of measurements.

Skill	Measurement	Description
Language skills (“Verbal fluency”)	Word generation (Nepsy II; Korkman et al., 2008) Sentence repetition (NEPSY II, Korkman et al., 2008)	Due to the broad scope of language skills, two related measurements were included in this aspect. (1) Word generation: a paper-based test involves mentioning as many food/animals related answers in 60 s as possible. (2) Sentence repetition, which is also related to auditory and linguistic short-term memory, is a paper-based test where the research assistant says one sentence in Finnish and the participant should repeat it back. The sentences get progressively longer and more complicated.
Inhibition	Flanker task (Eriksen and Eriksen, 1974; Roebers, 2017)	A shorter, playful version using fish figures was developed by this project researchers from the original task. The test was conducted on an iPad with a macro keyboard to select the direction of the fish.
Emotion recognition and Emotion naming	Teddy bear test (Kalland and Linnavalli, 2022)	In this paper-based test, a child is presented with 14 short story excerpts about a teddy bear and different scenarios. The child is first asked to choose which teddy bear drawing corresponds to the emotions of the story characters, and then verbally name that emotion. The complexity of emotions increases towards the end of the test. The story line consists of 36 items, but when more than 10 mistakes in a row were made, the remaining items were not calculated for the analysis.
Children’s musical perception skills	Musicality test (MMBB): music perception	The Centre of Excellence in Music, Mind, Body and Brain developed a battery of tasks targeting different musical tasks (Neto et al., 2025). In this study, a shortened version of the perception task based on “The Montreal Battery of Evaluation of Musical Abilities, MBEMA, on tablet” (Peretz et al., 2021) was used, as a measure of children’s music perception skills.
Children’s motor skills	Piilo test (Sääkslahti et al., 2021)	This test, developed in Finland, aims at providing a holistic picture of children’s motor skills. Done in groups of 2–3 children, with a maximum estimated duration of 30 min. The assessment enlarges static balance, dynamic balance, locomotor skills and object control skills.

### 2.4 Statistical analysis

A Generalized Linear Mixed modelling (GLMM) approach was used in a Bayesian framework. All analyses were carried out in the R environment through *brms* (Bürkner, 2017, 2018), *bayestestR* (Makowski et al., 2019a), and *modelbased* (Makowski et al., 2020). To inspect the role of our predictors on (1) Word Generation (WGS), (2) Sentence Repetition (SRS), (3) Music Perception (Mus_Per), (4) Motor Skills (PIILO), (5) Inhibition (Flanker) and (6) Emotion recognition skills (Teddy Bear; “TB”); we created GLMM models wherein these dependent variables (DVs) were predicted by previous attendance and at-home practices for both music and physical activity (refer to 2.3 Materials for a more detailed description). More precisely, the two-way interactions between previous attendance of music and PA-based extracurricular activities and at-home practices for PA and music were modelled. Finally, we modelled the two-way interactions between previous attendance of music and at-home musical practices and previous attendance of PA and at-home practices, hypothesizing that the effect of previous attendance might be different depending on the level of in-home practices.

In all models, the average over the caregivers’ education level and the participants’ age (in months) were added in a covariate perspective (i.e., to control for their effects). The linguistic skills (i.e., WGS and SRS) were modelled together in a multivariate model to account for their inherent correlation. In this model, the age at which the participants started to speak was added as a covariate. Given the link between emotion recognition and linguistic skills, WGS and SRS were added in the adjustment set of the Social–emotional skills model.

As a partial pooling technique, the ECEC was added as a random intercept to model their variability. All the dependent variables, predictors, and covariates were z-transformed prior to the modelling phase. Therefore, model coefficients for extracurricular activities represent the expected change in the outcome associated with a 1 SD-increase in months of attendance.

#### 2.4.1 Likelihood, prior distributions, and Bayesian indices

Consistent with a Generalized approach, we used different likelihood functions depending on the DVs. In particular, we used a Gaussian distribution for linguistic skills and music perception models. The flanker task and Teddy Bear scores were modelled with a skew-normal distribution (Azzalini, 2005). No link function was used in these models. Being bounded between zero and one, the PIILO variable was conveniently modelled with a Beta distribution and a logit link function.

In all models, we set regularizing (i.e., neutral) priors. More specifically, a zero-centered normal distribution with *SD* = 1 was employed for the intercept and beta coefficients, whereas the priors for the *SD* of the random intercept and sigma of the models were modelled with an exponential distribution with *λ* = 1 (McElreath, 2020). In the PIILO model, due to the link function, the zero-centered priors for the intercept and beta coefficients had *SD* = 1.5 (McElreath, 2020).

We used the Probability of Direction (*pd*) of the parameters as an index of the effect’s existence. Given the exploratory nature of the study, we considered as highly probable the effects whose parameters had *pd* > 90% (Makowski et al., 2019b). This metric was chosen because it has a direct relationship with the frequentist *p*-value (see Makowski et al., 2019b, p. 6). However, it is worth stressing that, different from the *p*-value, in the Bayesian framework, a Probability of Direction of 90% directly translates to a 90% probability that the effect exists (i.e., deviates from a null), given the data, with a consistent sign (either positive or negative).

Moreover, we resorted to the concept of ROPE (Region of Practical Equivalence) to assess the effect size (Kruschke, 2018; Makowski et al., 2019b); in particular, we computed how much of the 89% credible interval of the parameter distributions fell within the ROPE (for the rationale behind the 89% CI, see McElreath, 2020 and Kruschke, 2014). As we z-transformed the DVs, we set the ROPE to range between ± 0.10, i.e., a negligible effect.

For each parameter, we reported the Maximum A Posteriori (MAP) as the centrality measure and the 89% equal-tailed Credible Intervals to show the uncertainty of the estimates.

A detailed description of the posterior distributions of the parameters of all models is available in the Supplementary materials. These materials also report the conditional and marginal Bayesian *R*^2^ (Gelman et al., 2019) of all models computed using the *performance* package (Lüdecke et al., 2021), as well as a visual Posterior Predictive Check (PPC) in the form of density overlay plots.

## 3 Results

### 3.1 Demographics

Table 2 describes the main demographic information from the participants as gathered in the questionnaires filled in by the caregivers.

**Table 2:** Demographics information.

Variable	Response options classified	N	%
Gender	Female	55	53.90%
	Male	45	44.10%
	Prefer not to say	1	1.00%
	Missing	1	1.00%
Age started speaking	Before 12 months	1	1.00%
	Between 12 and 23 months	48	47.00%
	Between 24 and 35 months	43	42.00%
	From 36 and 42 months	3	3.00%
	Missing	7	7.00%
Caregiver 1 education level ^a^	Less than bachelor’s degree	22	21.60%
	Bachelor’s degree or equivalent	37	36.30%
	Master’s degree or more	42	41.20%
	Missing	1	1.00%
Caregiver 2 education level ^a^	Less than bachelor’s degree	35	35.00%
	Bachelor’s degree or equivalent	29	29.00%
	Master’s degree or more	35	35.00%
	Missing	2	1.00%
Hobbies attendance	Yes	54	52.90%
	No	48	47.10%
Music extracurricular attendance	0 months	64	62.70%
	1–9 months	22	21.60%
	10–37 months	15	14.70%
	Missing	1	1.00%
Sports hobbies attendance	0 months	60	58.80%
	1–9 months	26	25.50%
	10–37 months	16	15.70%
Singing together	No	11	10.80%
	1/month	9	8.80%
	1/week	28	27.50%
	Several days /week	24	23.50%
	Everyday	29	28.40%
	Missing	1	1.00%
Playing instruments together ^b^	No	38	37.30%
	1/month	33	32.40%
	1/week	25	24.50%
	Several days /week	5	4.90%
	Everyday	0	
	Missing	1	1.00%
Low intensity exercise ^c^	No	0	
	1/month	1	1.00%
	1/week	21	20.60%
	Several days /week	27	26.50%
	Everyday	51	50.00%
	Missing	2	2.00%
High intensity exercise ^d^	No	4	3.90%
	1/month	15	14.70%
	1/week	36	35.30%
	Several days /week	37	36.30%
	Everyday	8	7.80%
	Missing	2	2.00%

a For analysis, the average of both caregivers’ education level was used (in cases where there was only one caregiver, then this was taken as a full value).

b E.g., tapping, clapping….

c Low intensity refers to light activities such as walking, rocking and balancing.

d High intensity: brisk and fast-paced exercise such as cycling, trampoline jumping, skiing….

The majority of music-related extracurricular activities were music playschool (“muskari” in Finnish) which refers to a common activity in Finland for early childhood years, and usually consists of music and movement, playful activities with the goal of letting the children familiarize with music. Physical activity-based activities were, however, more diverse, the most common ones being swimming, athletics, and football. Table 3 shows the commonalities between the attendance and length categories.

**Table 3:** Crosstabs of extracurricular activities attendance.

Music attendance		Sports attendance			
		0 months	1–9 months	10–37 months	Total
	0 months	40	17	7	64
	1–9 months	14	3	5	22
	10–37 months	5	6	4	15
Total		59	26	16	101

### 3.2 Modelling

#### 3.2.1 Word Generation Score (WGS)

In the WGS part of the linguistic skills model, the parameter of the attendance of music extracurricular activity showed a small but 92.09% probable positive association, (*β* = 0.16, 89% CI [−0.02, 0.35], *pd* = 92.09%, *ROPE%* = 27.62), suggesting moderate directional evidence for a positive small effect.

**Figure 1:**
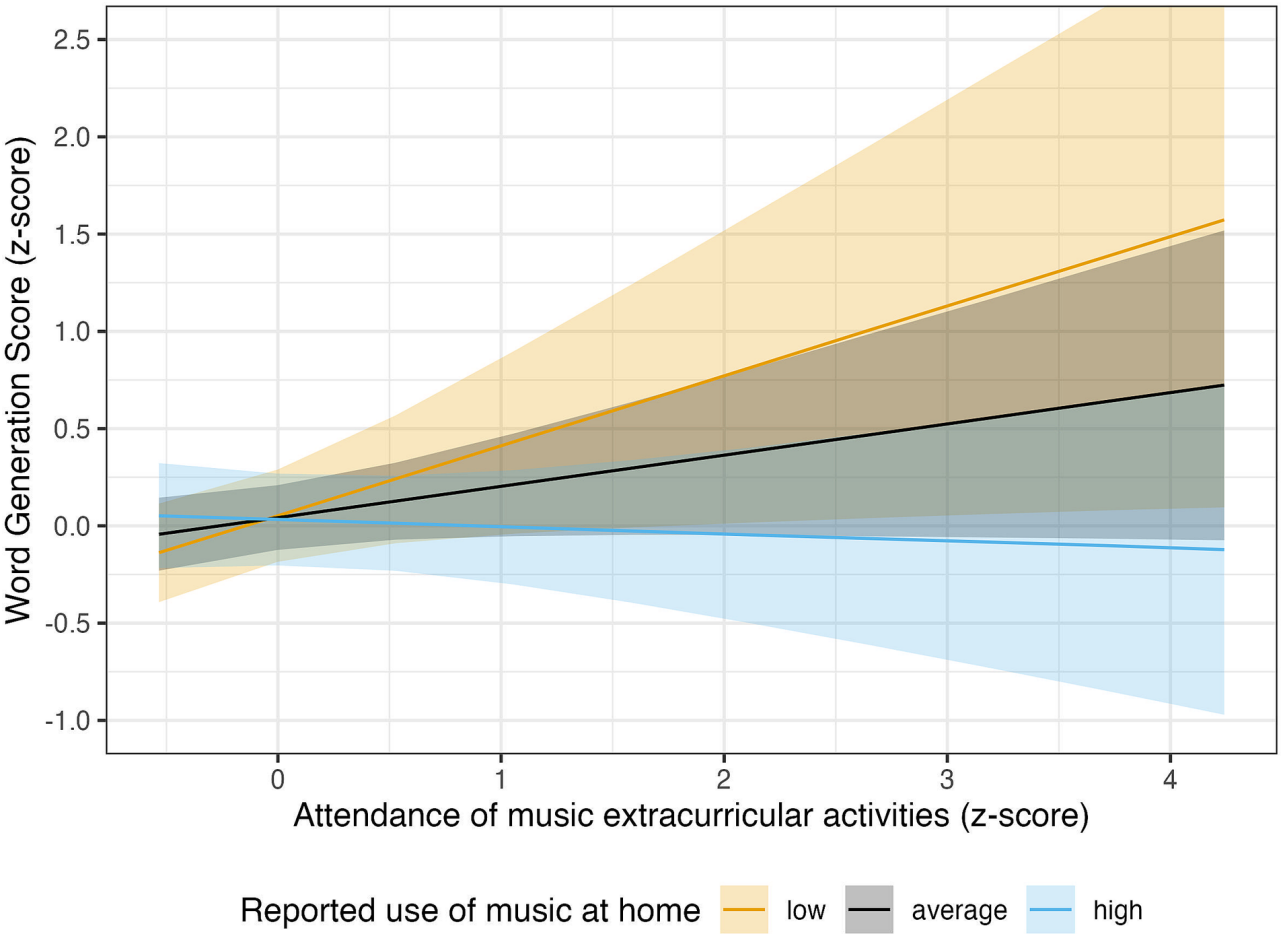
The association between attendance of music extracurricular activities and WGS was maximal for the children with the lowest amount of at-home music practice (in orange: *β* = 0.36, 89% CI [0.03, 0.69], *pd* = 95.83%, *ROPE%* = 8.72%), smaller for kids with an average amount of such practices (in black: *β* = 0.16, 89% CI [−0.02, 0.34], *pd* = 91.96%, *ROPE%* = 28.44) and absent for kids with a lot of at-home music practice (in light-blue: *β* = −0.04, 89% CI [−0.24, 0.1], *pd* = 61.62%, *ROPE%* = 59.01). The solid lines show the posterior MAP of the predicted values, and the shaded regions represent the 89% credible intervals, quantifying uncertainty around the estimates.

Its interaction with the at-home music practices was also relevant, *β* = −0.19, 89% CI [−0.40, 0.01], *pd* = 93.96%. To clarify, ROPE percentages are not reported for interaction parameters because these capture differences of differences, which do not lend themselves to a direct interpretation in terms of practical equivalence. When extracting the estimated marginalized slopes, we noticed that the positive association was maximal for the children with the lowest amount of at-home music practice (*β* = 0.36, 89% CI [0.03, 0.69], *pd* = 95.83%, *ROPE%* = 8.72%), smaller for kids with an average amount of such practices (*β* = 0.16, 89% CI [−0.02, 0.34], *pd* = 91.96%, *ROPE%* = 28.44) and absent for kids with a lot of at-home music practice (*β* = −0.04, 89% CI [−0.24, 0.1], *pd* = 61.62%, *ROPE%* = 59.01) (Figure 1).

**Figure 2:**
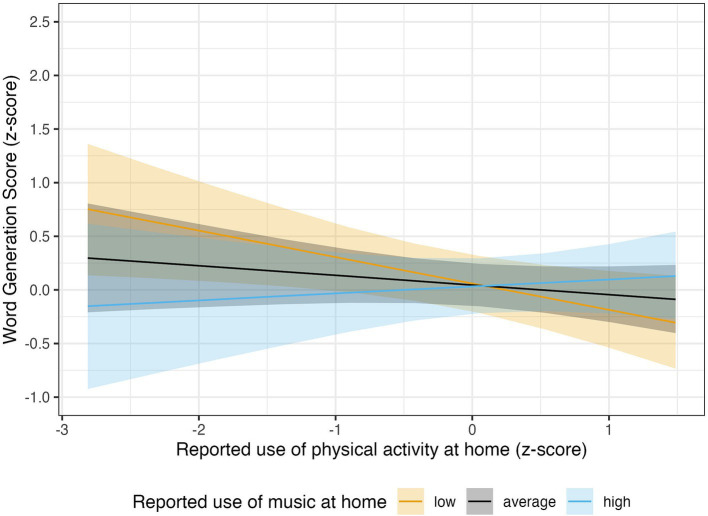
The association between reported use of physical activity at home and WGS was likely existent only for low values of at-home musical practices (in orange: *β* = −0.25, 89% CI [−0.45, −0.04], *pd* = 97.06%, *ROPE%* = 10.79). For average (in black: *β* = −0.09, 89% CI [−0.25, 0.07], *pd* = 81.89%, *ROPE%* = 52.65) and especially high values (in light-blue: *β* = 0.06, 89% CI [−0.18, 0.30], *pd* = 66.29%, *ROPE%* = 47.91), the evidence for the association was very scarce. The solid lines show the posterior MAP of the predicted values, and the shaded regions represent the 89% credible intervals, quantifying uncertainty around the estimates.

We also found a highly probable interaction between music and PA at-home engagement, *β* = 0.15, 89% CI [0.00, 0.31], *pd* = 94.34%. The analysis of the marginalized slopes showed that the negative association between at-home physical activity and WGS was likely existent only for low values of at-home musical practices, *β* = −0.25, 89% CI [−0.45, −0.04], *pd* = 97.06%, *ROPE%* = 10.79. For average and especially high values, the evidence for the association was very scarce, *pd* = 81.89 and 66.29%, respectively (Figure 2).

#### 3.2.2 Sentence Repetition Score (SRS)

**Figure 3:**
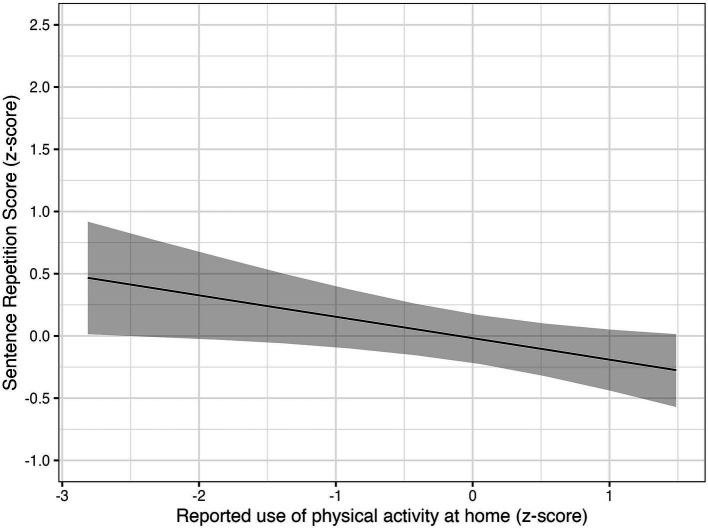
The association between reported use of physical activity at home and SRS was very probable, even though small in size (*β* = −0.17, 89% CI [−0.32, −0.02], *pd* = 96.86%, *ROPE%* = 20.33%). The solid line shows the posterior MAP of the predicted values, and the shaded region represents the 89% credible intervals, quantifying uncertainty around the estimates.

In the SRS part of the model, we noticed a negative association with the at-home physical activity practices, *β* = −0.17, 89% CI [−0.32, −0.02], *pd* = 96.86%, *ROPE%* = 20.33% (Figure 3).

Finally, the correlation between the Word Generation and Sentence Repetition scores was moderate and certain, *r* = 0.38, 89% CI [0.22, 0.52], *pd* = 99.97%, *ROPE%* = 0.

#### 3.2.3 Inhibition (Flanker Task)

None of the parameters of the effects of interest showed a straightforward probability of direction except for the interaction between attendance to music and physical extracurricular activity, *β* = 0.16, 89% CI [−0.03, 0.36], *pd* = 91.27%. However, none of the marginalized slopes showed a straightforward direction, ranging from *pd* = 54.90 to 88.92%.

#### 3.2.4 Emotion Recognition Skills (teddy bear)

**Figure 4:**
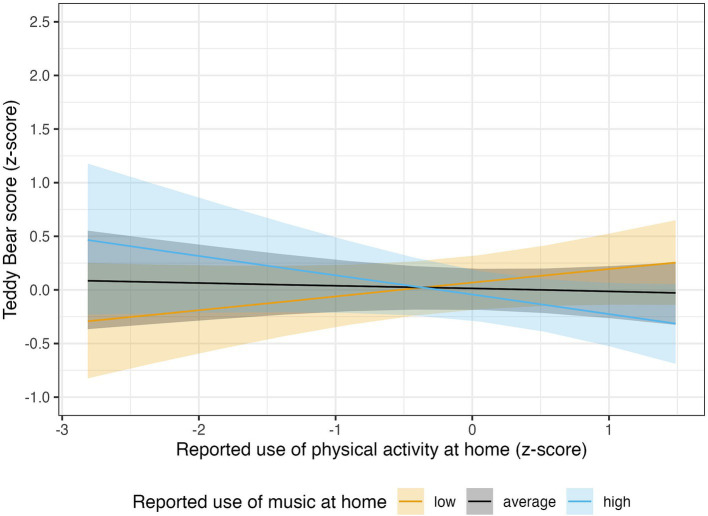
The association between reported use of physical activity at home and the Teddy Bear score was tendentially negative only for the participants who engaged in a high amount of music at home (in light-blue: *β* = −0.18, 89% CI [−0.41, 0.04], *pd* = 90.57%, *ROPE%* = 26.79). In participants with a low level of at-home musical practices, at-home PA engagement seems to be associated with a slight improvement in the Teddy Bear score, although the evidence for the existence of this association is scarcer (in orange: *β* = 0.13, 89% CI [−0.06, 0.31], *pd* = 87.08%, *ROPE%* = 40.43). The solid lines show the posterior MAP of the predicted values, and the shaded regions represent the 89% credible intervals, quantifying uncertainty around the estimates.

An interesting interaction effect between the two at-home practices emerged in the Teddy Bear model, *β* = −0.15, 89% CI [−0.29, −0.01], *pd* = 96.09%. The nature of such an interaction can be better grasped from Figure 4. When computing the marginalized slopes, we understand that at-home PA practices are likely to be negatively associated with the Teddy Bear score only when the participants engage in a high amount of music at home, *β* = −0.18, 89% CI [−0.41, 0.04], *pd* = 90.57%, *ROPE%* = 26.79. Conversely, if they engage in a low level of at-home musical practices, at-home PA engagement seems to be associated with an improvement in the Teddy Bear score, although the evidence for the existence of this association is scarcer, *β* = 0.13, 89% CI [−0.06, 0.31], *pd* = 87.08%, *ROPE%* = 40.43.

As expected, both WGS and SRS covariates showed positive associations, *β* = 0.25, 89% CI [0.06, 0.44], *pd* = 98.41%, *ROPE%* = 5.48 and *β* = 0.43, 89% CI [0.19, 0.61], *pd* = 99.93%, *ROPE%* = 0, respectively.

#### 3.2.5 Music Perception and Motor Skills

In the music perception and motor skills models, none of the parameters of the effects of interest had *pd* > 90%.

## 4 Discussion

Gaining a deeper understanding of how to support children’s development during the early childhood years is extremely important, as this period has a key influence on later development. The present exploratory study investigated whether participating in music and physical activity-based extracurricular activities as well as the music and PA activities at home had any association with children’s verbal fluency, emotion recognition, inhibition, music and motor skills. In our data, we observed some potential trends regarding verbal fluency scores: (1) a small positive association between music-related extracurricular activities and the word generation score, and (2) a negative association between the physical activity at home and the sentence repetition score. Moreover, an opposing interaction effect of physical and musical activity at home together with the score of the teddy bear test (emotion recognition skills) was also noted. No other associations were observed between the activities and the rest of the measured skills. The implications of these findings are discussed in line with previous results and future suggestions.

### 4.1 Music and physical activity engagement in extracurricular activities

In our data, we observed a small positive association between extracurricular music attendance and word generation scores. While the connection between music and language development has previously been explored (Martin-Martinez et al., 2023; Pino et al., 2023), the specific evidence on verbal fluency skills has been limited, and our finding, although suggestive, might provide more clarity to this. However, such association was not observable with our other language-related measure sentence repetition. The sentence repetition score is also connected to auditory and linguistic short-term memory (Korkman et al., 2008), suggesting that in order to support the maturation of the kind of cognitive processing needed in this task, a factor—such as music engagement—should also support the development of working memory.

Language development has been linked with social–emotional aspects. Hanno and Surrain (2019) argue that language facilitates creating mental representations and constructs about oneself and others. In a previous study, the same emotion recognition test as used in this study (The Teddy Bear Test) was found to be associated with children’s language skills (Kalland and Linnavalli, 2022). While this association was also found in our emotion recognition skills model, no other association with music or PA extracurricular attendance was observed. The heterogeneity of tasks for studying children’s social–emotional development has already been flagged (Blasco-Magraner et al., 2021), as for example, free-label emotion recognition tasks are more challenging than match-to-sample ones (Riddell et al., 2024). Still, as our focus was also on language development and its association impact with other aspects of children’s development, the language-dependent Teddy Bear test was included in the test battery.

No other associations were observed regarding the rest of the skills measured. Previous studies have reported an improvement in inhibitory control from music and PA engagement (Jamey et al., 2024; Wang et al., 2023), but the strongest evidence comes from longer and longitudinal engagement (De Greeff et al., 2018; Morales et al., 2024). In the present study, the wide variety in regularity, duration and types of children’s extracurricular activities (especially in PA), may explain that the results are not in line with previous studies. Structured engagement in music and PA extracurricular activities did not have a direct association with the music and motor skills measured, which might relate to the age appropriateness of the used measures. Another factor is that music and motor skills are intertwined with a wide variety of other aspects and skills which might require a longer time to consolidate.

When discussing the benefits of engagement in extracurricular activities, it is important to acknowledge the inequality and accessibility challenges for some populations and specific social contexts. Although participation in sports hobbies in Finland has increased, some regional and socioeconomic differences remain significant (Lounassalo et al., 2025). Related to this, more than half of the children in our sample did not have any previous attendance in extracurricular activities. It is unclear whether this suggests some accessibility limitations but in any case, emphasizes the importance of providing more flexible and inclusive options.

### 4.2 Music and physical activity engagement at home

Although the home environment has a central role in children’s development, to the authors’ knowledge, the impact of music and physical activity engagement at home has not been widely studied. Whilst some large-scale studies are available (Kostilainen et al., 2024.; Papadimitriou et al., 2021., Williams et al., 2015), except for Politimou et al. (2019), the approach has typically focused on retrospectively asking about music and PA engagement and their association on later development.

Contrary to the positive correlation between music extracurricular participation and word generation score, we observed an opposite association between sentence repetition (the other verbal fluency test used) and the reported use at home of physical activity. An interaction was also observable for the group with the lowest reported use of music at home. Previous evidence suggests a positive association of physical activity and preschoolers’ language skills (Kamelia et al., 2025; Mulé et al., 2022). It is true that specifically for verbal fluency skills, the evidence is more limited (Martin-Martinez et al., 2023), with positive contributions being associated more with the pedagogical approach rather than the amount of physical activity (Olive et al., 2024).

Still, when discussing the at-home context, contextual and relational factors such as parent–child interactions and the quality of the home learning environment are central in supporting language development (Brushe et al., 2025; Lovčević, 2025). Moreover, the use of language varies within families just as much as between families (d'Apice et al., 2019), supporting the view that language development is not only input dependent but also contextually constructed, with socioeconomic conditions playing a role in parent–child interactions and language learning (Attig and Weinert, 2020; Madigan et al., 2019). The parental language interaction during physical activity with the child probably varies between families, which could also act as a cofounding variable. As the current data is based on cross-sectional self-reports by caregivers, this unexpected result might thus be more related to the unaccounted contextual and cofounding factors mentioned, and this challenge should be considered in future research.

A small positive interaction between music extracurricular engagement and use at home for word generation score was also observed. This finding is somewhat in line with Politimou et al. (2019), who found that the interaction between musical skills and the reported music experience at home was predictive of 3-4-year-olds’ language development. Music exposure at home has indeed been associated with quality shared reading interactions, with parent self-efficacy influencing this correlation (Liu et al., 2024). However, the trend observed here was the opposite for those participants with the most frequent use of music at home. Some possible explanations could be that the current study excluded other musical skills such as singing production and accuracy, information on more passive exposures such as music listening (considered by Politimou et al., 2019); and did not gather other mediating aspects such as parental self-efficacy as Liu et al. (2024).

Another unexpected result was the interaction effect observed between the highest amount of music and PA reported and the lowest scores in the Teddy Bear Test (emotion recognition skills). This test seems to be dependent on language skills, and opposite trends were observed between music/PA engagement in verbal fluency scores. The Teddy Bear test takes typically about 15 min with this age group, requiring sustained attention levels and an ability to stay still for a long time. In fact, this test was typically done after the language-based measurements, which might have caused some test and concentration fatigue. Individual differences such as the fact that children with the highest use of music and PA at home might be used to having more active and hands-on activities might have contributed to this unexpected tendency. Relying on caregivers’ self-reports might also cause some limitations for ecological validity and reliability. These types of measures capture more quantity than quality, posing also challenges to estimate the frequency of common behaviors in a long period of time (Morsbach and Prinz, 2006). They might also reveal an over-representation of desirable practices of what is considered as “good parenting” (Cates et al., 2023), as well as limited convergent validity with other types of direct measures (Li et al., 2019). Further research should consider all these aspects to explore this trend deeper.

No other association between inhibitory control, musical, or motor skills was observed. One potential reason is the large frequency difference between the questioned practices: in our data, the frequency of singing compared to playing an instrument was quite different. A similar trend between low intensity and high intensity exercise appeared (50% vs. 7.8%), emphasizing the need to be even more detailed in the questions of self-reported activities in the future.

While previous research has indeed provided some stronger support for the association of children’s early musical experience and some of the aspects studied here, to the authors’ knowledge, it has been mainly the prediction value of participation during early childhood with development later in childhood. For example, Williams et al. (2015) found a significant association between early children’s musical context and their later prosocial and emotional regulation skills, features of social–emotional development differing from the emotion recognition skills measured in the present study. Regarding musical skills, musical experience at home has only seemed to influence children’s singing in tune ability (Politimou et al., 2019), a skill which was not considered in our study.

In regard to physical activity engagement, the home environment is crucial for later development of fundamental motor skills (Barnett et al., 2019). Time spent outdoors has also been associated with physical activity, especially among girls (Kwon et al., 2022). Indeed, in a Finnish cohort, Luukkainen et al. (2025) found that time spent outdoors as reported by caregivers, predicted only girls’ later higher scores in jumping sideways, object control, and fundamental motor skills. This literature suggests that the effects from activities in early ages in development might only be fully observable after several years, as the maturation of complex skills involves several aspects of development. In a longitudinal study, children’s (mean age of 6.26 yrs) self-perceived motor competence (PMC) predicted both actual and perceived motor competence in later ages (mean age 8.76 yrs), with an interesting gender difference of boys overestimating their PMC more than girls (Niemistö et al., 2023). This finding thus supports how physical self-concept seems to also strongly relate to physical activity (Meklin et al., 2024), including children’s own self-concept in the longitudinal analysis of the project.

### 4.3 Limitations and future directions

The present results suggest some limitations as the attendance in extracurricular activities was skewed in our study and more than half of the participants had not attended such activities at all, which limits the robustness of the statistical analysis. Moreover, whether this reflects accessibility and inequality issues should be explored further, which emphasizes the key relevance of the at-home context and practices in this age group. The present study also highlights the challenges with the age appropriateness of the measurements and skills studied. They involve many different developmental aspects, some of which may demand longer exposure to music and PA activities, and take longer time to develop. However, as the study is part of a longitudinal design, the measures were planned to reflect the development of the children, even though it might involve some challenges at the beginning of the follow-up. While gathering caregivers’ self-reports is easier than conducting home observations (Cates et al., 2023), these type of measures also present limitations, as previously discussed (see Section 4.2.). Most importantly, the cross-sectional and observational nature of the current study prevents any causality claims, and calls for caution in interpreting the results. However, the inconclusive results in this study and the mixed reports in previous literature emphasize the need for future studies in how to best support children’s diverse aspects of development.

## 5 Conclusion

To conclude, our findings suggest that extracurricular music activities as well as the interaction with at-home use of music may have a positive association on children’s language skills, specifically on verbal fluency, which is in line with previous research. Interestingly, an opposite trend was observed between at-home practice of physical activity and sentence repetition scores. Higher engagement in music and PA at home revealed a weak decreasing tendency with the scores of an emotion-recognition test. No other associations on the rest of the measured skills were observed. The cross-sectional nature of this study prevents from making any causal or developmental effects, which might also reflect underlying factors not considered in this study. Individual differences may be one possible explanation for the associations we observed in our findings. Previously, for instance, Niemistö et al. (2023) found that individual differences seem to be the most important predictors of motor competence in a similar age group. The effects of music or PA activities on children’s different skills might also take more time to develop, thus the effect might only be observable later in childhood (Williams et al., 2015), and children’s emotion recognition skills seem to improve with age (Riddell et al., 2024). Other contextual and situational factors that were not reflected in this study’s measurements, should also be considered in further research. On balance, these findings show the complexity of child development and the role of early experiences both at-home and in other learning environments, showing how several skills and factors might interconnect with the learning associations happening. This should be more thoroughly considered in future longitudinal studies.

## Data Availability

The raw data supporting the conclusions of this article will be made available by the authors, without undue reservation.
