# Supplementary material for: Music and physical activity in early childhood: the ambiguous role of the at-home context and extracurricular activities
Source: Front Psychol. 2026 Feb 12;17:1729705. doi: 10.3389/fpsyg.2026.1729705 (PMC12935927; doi:10.3389/fpsyg.2026.1729705)
Supplement: Supplementary file 1 [file Data_Sheet_1.pdf]

Supplementary Materials

Supplementary Materials for:

Lucendo-Noriega, A., Sääkslahti, A., Ansani, A., Henttonen, K., Carlson, E., Saarikallio, S.H., Toiviainen, P., & Linnavalli, T. (2026). Music and physical activity in early childhood: the ambiguous role of the at-home context and extracurricular activities. *Frontiers in Psychology*, 17:1729705

Table of Contents

1. Posterior summaries *2*
- 1.1. Linguistic Skills (Multivariate Model) 2
- 1.2. Flanker Task 4
- 1.3. Teddy Bear 5
- 1.4. Music Perception 6
- 1.5. PIILO 7
- 1.6. Legend 7

2. Model performance *8*
- 2.1. Bayesian R^2 8
- 2.1. Posterior Predictive Check (PPC) 9

3. References *10*

1. Posterior summaries

In the following, we describe the posterior distributions of the parameters of all models. For all parameters, the reader will find: the Maximum A Posteriori as the central tendency measure (*MAP*), 89% equal-tailed Credible Intervals (*89% LB* and *89% UB*), Probability of Direction (*pd*), Percentage of the posterior distribution in the Region Of Practical Equivalence (*ROPE%*), and the Effective Sample Size (*ESS*). Parameters of focal predictors whose *pd* > 90% are indicated **in bold**. As we z-transformed the DVs, we set the ROPE to range between ± 0.10, i.e., a negligible effect according to Cohen (1988). In particular, we computed how much of the 89% of the parameter distributions fell within the ROPE (for the rationale behind the 89% CI, see McElreath, 2020 and Kruschke, 2014). For a description of all indices, see Makowski and colleagues (2019).

Consistent with a generalised approach, we used different likelihood functions depending on the DVs. In particular, we used a Gaussian distribution for linguistic skills and music perception models (i.e., Models 1 and 4). The Flanker task and Teddy Bear scores (i.e., Models 2 and 3) were modelled with a skew-normal distribution (Azzalini, 2005). No link function was used in these models. Being bounded between zero and one, the PIILO variable was conveniently modelled with a Beta distribution and a logit link function (i.e., Model 5).

For all the details about prior distributions of the model, please refer to the paper.

1.1. Linguistic Skills (Multivariate Model)

DV	Parameter	MAP	89% LB	89% UB	pd	ROPE%	ESS
WGS	Intercept	0.02	-0.16	0.23	60.32	63.86	22307.54
WGS	PA_Activity	0.03	-0.14	0.19	60.06	72.59	30755.85
WGS	**MUS_Activity**	**0.16**	**-0.02**	**0.35**	**92.09**	**27.62**	**24398.58**
WGS	PA_Use	-0.08	-0.25	0.07	81.03	53.34	28344.79
WGS	MUS_Use	-0.01	-0.18	0.16	53.66	75.39	29106.15
WGS	Caregiver Avg. Education	-0.00	-0.19	0.17	53.36	71.08	27051.06

DV	Parameter	MAP	89% LB	89% UB	pd	ROPE%	ESS
WGS	Month born	-0.30	-0.47	-0.15	99.88	0.00	28469.33
WGS	Speaking Age	-0.25	-0.42	-0.09	99.20	1.18	30362.65
WGS	PA_Activity:MUS_Activity	-0.07	-0.25	0.10	75.75	60.15	26815.95
WGS	**PA_Use:MUS_Use**	**0.15**	**0.00**	**0.31**	**94.34**	**26.04**	**27711.36**
WGS	PA_Activity:PA_Use	0.02	-0.11	0.15	59.37	89.24	30787.30
WGS	**MUS_Activity:MUS_Use**	**-0.19**	**-0.40**	**0.01**	**93.96**	**19.41**	**24617.17**
SRS	Intercept	-0.01	-0.22	0.17	56.57	68.05	18242.02
SRS	PA_Activity	-0.10	-0.25	0.04	87.42	29.20	25552.15
SRS	MUS_Activity	-0.03	-0.18	0.14	57.75	68.21	24324.53
SRS	**PA_Use**	**-0.17**	**-0.32**	**-0.02**	**96.86**	**20.33**	**27717.94**
SRS	MUS_Use	0.11	-0.04	0.26	88.87	41.95	28252.89
SRS	Caregiver Avg. Education	0.28	0.11	0.42	99.58	1.45	27341.87
SRS	Month born	-0.12	-0.27	0.01	92.49	50.15	29382.18
SRS	Speaking Age	-0.44	-0.58	-0.29	100.00	0.00	29192.32
SRS	PA_Activity:MUS_Activity	0.10	-0.06	0.26	84.47	73.70	25309.15
SRS	PA_Use:MUS_Use	0.03	-0.10	0.18	68.15	85.33	26921.19
SRS	PA_Activity:PA_Use	-0.00	-0.12	0.10	53.06	88.57	29734.84
SRS	MUS_Activity:MUS_Use	0.05	-0.13	0.23	68.62	66.33	23440.28

1.2. Flanker Task

Parameter	MAP	89% LB	89% UB	pd	ROPE%	ESS
Intercept	0.00	-0.20	0.20	50.69	65.02	24261.07
PA_Activity	0.01	-0.17	0.18	54.62	72.47	34278.05
MUS_Activity	0.11	-0.10	0.28	79.97	47.91	24827.57
PA_Use	-0.02	-0.18	0.18	51.29	71.58	39656.62
MUS_Use	0.07	-0.10	0.22	73.42	67.04	36626.24
Caregiver Avg. Education	0.14	-0.05	0.32	87.36	38.77	31724.67
Month born	-0.09	-0.25	0.08	77.86	59.30	37651.45
**PA_Activity:MUS_Activity**	**0.16**	**-0.03**	**0.36**	**91.27**	**27.37**	**26454.30**
PA_Use:MUS_Use	0.04	-0.13	0.19	60.88	74.45	36946.10
PA_Activity:PA_Use	-0.05	-0.20	0.07	76.25	70.54	40726.85
MUS_Activity:MUS_Use	-0.12	-0.32	0.10	79.53	46.74	24776.85

1.3. Teddy Bear

Parameter	MAP	89% LB	89% UB	pd	ROPE%	ESS
Intercept	-0.01	-0.22	0.16	57.53	69.19	16366.96
PA_Activity	0.00	-0.15	0.16	51.09	79.79	21128.20
MUS_Activity	0.12	-0.05	0.29	87.66	41.26	20978.36
PA_Use	-0.03	-0.17	0.12	61.22	79.27	25721.24
MUS_Use	-0.05	-0.20	0.09	72.95	70.62	26075.69
Caregiver Avg. Education	-0.07	-0.25	0.10	74.77	60.83	22965.90
Month born	-0.08	-0.25	0.09	77.25	57.98	18256.03
WGS	0.25	0.06	0.44	98.41	5.48	19917.20
SRS	0.43	0.19	0.61	99.93	0.00	15080.94
PA_Activity:MUS_Activity	-0.03	-0.20	0.14	61.16	72.53	22769.15
**PA_Use:MUS_Use**	**-0.15**	**-0.29**	**-0.01**	**96.09**	**23.84**	**23068.89**
PA_Activity:PA_Use	-0.03	-0.14	0.08	67.75	87.63	27402.96
MUS_Activity:MUS_Use	-0.03	-0.21	0.16	59.74	67.42	19573.29

1.4. Music Perception

Parameter	MAP	89% LB	89% UB	pd	ROPE%	ESS
Intercept	-0.02	-0.23	0.18	58.20	64.39	18358.85
PA_Activity	-0.08	-0.25	0.09	77.77	58.83	33658.99
MUS_Activity	-0.05	-0.23	0.13	65.66	65.56	28087.24
PA_Use	-0.03	-0.23	0.10	72.25	66.53	35838.78
MUS_Use	-0.03	-0.21	0.13	64.62	70.71	36570.22
Caregiver Avg. Education	0.08	-0.10	0.25	75.81	59.27	30353.70
Month born	-0.00	-0.16	0.16	50.20	77.50	39929.58
PA_Activity:MUS_Activity	-0.13	-0.31	0.05	88.20	36.67	30849.27
PA_Use:MUS_Use	-0.08	-0.22	0.09	75.73	64.36	34278.47
PA_Activity:PA_Use	0.10	-0.02	0.23	89.58	48.00	37258.90
MUS_Activity:MUS_Use	0.06	-0.14	0.26	69.09	58.69	27441.22

1.5. PIILO

Parameter	MAP	89% LB	89% UB	pd	ROPE%	ESS
Intercept	0.03	-0.16	0.21	58.24	70.46	14495.82
PA_Activity	0.11	-0.04	0.27	88.76	42.66	35789.31
MUS_Activity	0.05	-0.13	0.21	66.87	68.12	32351.31
PA_Use	0.10	-0.04	0.25	88.32	46.55	32197.48
MUS_Use	0.04	-0.11	0.19	68.45	73.58	27050.85
Caregiver Avg. Education	-0.11	-0.26	0.04	88.25	44.14	33408.45
Month born	-0.21	-0.35	-0.06	98.67	8.24	37583.65
PA_Activity:MUS_Activity	-0.03	-0.18	0.16	56.19	74.06	30553.06
PA_Use:MUS_Use	-0.06	-0.20	0.07	77.82	68.73	35707.99
PA_Activity:PA_Use	0.03	-0.08	0.15	66.48	86.40	33028.29
MUS_Activity:MUS_Use	0.05	-0.16	0.25	62.96	61.59	30189.75

1.6. Legend

- *PA_Activity*: Physical Activity-based extracurricular activity
- *MUS_Activity*: Music-based extracurricular activity
- *PA_Use*: Reported use of at-home Physical Activity
- *MUS_Use*: Reported use of at-home Musical Activity
- *Caregiver Avg. Education*: Average level of Education of both caregivers
- *Month born*: Month of birth
- *Speaking Age*: Age at which the kid started to speak

- *WGS*: Word Generation Score
- *SRS*: Sentence Repetition Score

2. Model performance

2.1. Bayesian R^2

Conditional and Marginal Bayesian R^2 (Gelman et al., 2019) have been computed via the `r2_bayes` function of the *performance* package (Lüdecke et al., 2021).

DV	Marginal R^2 [89% LB, UB]	Conditional R^2 [89% LB, UB]
Word Generation Score (WGS)	.273 [.179, .365]	.291 [.194, .384]
Sentence Repetition Score (SRS)	.369 [.280, .459]	.397 [.299, .489]
Flanker Task	.154 [.077, .233]	.165 [.086, .246]
Teddy Bear	.390 [.212, .536]	.419 [.248, .559]
Music Perception	.163 [.079, .242]	.199 [.106, .290]
PIILO	.195 [.110, .278]	.220 [.131, .312]

2.1. Posterior Predictive Check (PPC)

For each dependent variable, the black line represents the observed distribution of the data, while the light-blue lines correspond to 300 random replicated posterior distributions of the fitted models. The figure illustrates that the models adequately reproduce the overall shape, central tendency, and dispersion of the observed outcomes, with no major systematic discrepancies between the observed distributions and the posterior predictive ones.

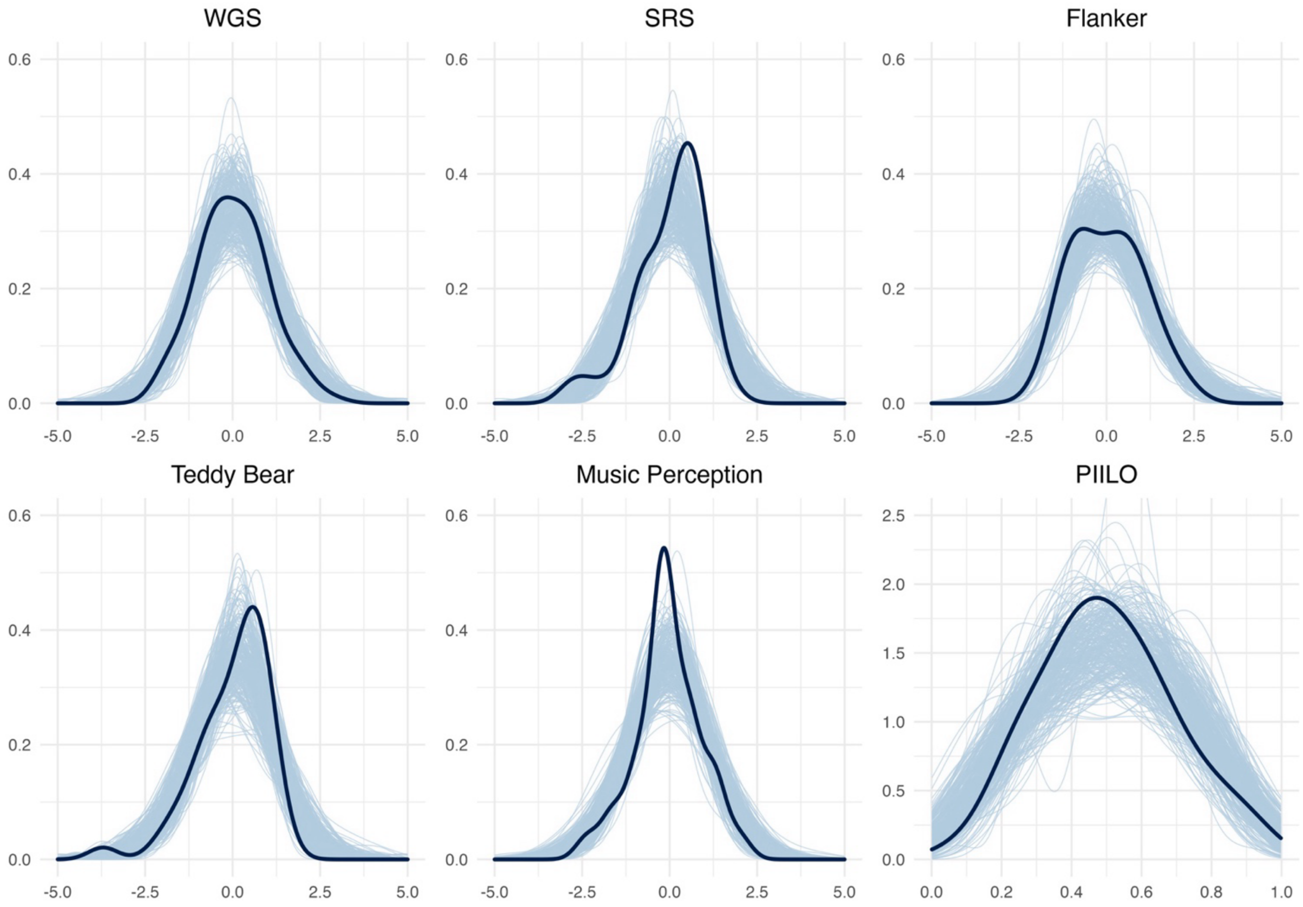

3. References

Azzalini, A. (2005). The skew-normal distribution and related multivariate families. *Scandinavian Journal of Statistics, 32*(2), 159-188. https://doi.org/10.1111/j.1467-9469.2005.00426.x

Cohen, J. (1988) *Statistical power analysis for the behavioral sciences* (2nd ed.). Hillsdale, NJ: Erlbaum.

Gelman, A., Goodrich, B., Gabry, J., & Vehtari, A. (2019). R-squared for Bayesian Regression Models. *The American Statistician*, *73*(3), 307–309. https://doi.org/10.1080/00031305.2018.1549100

Kruschke, J. (2014). *Doing bayesian data analysis: A tutorial with R, JAGS, and Stan*. Academic Press, Burlington, MA.

Lüdecke, D., Ben-Shachar, M., Patil, I., Waggoner, P., & Makowski, D. (2021). performance: An R Package for Assessment, Comparison and Testing of Statistical Models. *Journal of Open Source Software*, *6*(60), 3139. https://doi.org/10.21105/joss.03139

Makowski, D., Ben-Shachar, M.S., Chen, S.H.A., Lüdecke, D. (2019). Indices of Effect Existence and Significance in the Bayesian Framework. *Frontiers in Psychology,* 10, 2767. https://doi.org/10.3389/fpsyg.2019.02767

McElreath, R. (2020). *Statistical rethinking: A Bayesian course with examples in R and Stan* (Second edition). Chapman and Hall/CRC, New York, New York, USA.
